# Candida albicans biofilms on different materials for manufacturing implant abutments and prostheses

**DOI:** 10.4317/medoral.23157

**Published:** 2019-12-24

**Authors:** Asier Eguia, Ane Arakistain, Iker De-la-Pinta, José López-Vicente, Elena Sevillano, Guillermo Quindós, Elena Eraso

**Affiliations:** 1 Departamento de Estomatología II, Facultad de Medicina y Enfermería, Universidad del País Vasco/Euskal Herriko Unibertsitatea UPV/EHU, Bilbao, Spain; 2 Departamento de Inmunología, Microbiología y Parasitología, Facultad de Medicina y Enfermería, Universidad del País Vasco/Euskal Herriko Unibertsitatea UPV/EHU, Bilbao, Spain

## Abstract

**Background:**

Morphological, physical and chemical properties of both implants and prostheses can determine the biofilm formation on their surface and increase the risk of biological complications. The aim of this study was to evaluate the capacity of biofilm formation of *Candida albicans* on different materials used to manufacture abutments and prostheses.

**Material and Methods:**

Biofilm formation was analyzed on cp grade II titanium, cobalt-chromium alloy and zirconia, silicone, acrylic resin (polymethylmethacrylate) and nano-hybrid composite. Some samples were partially covered with lithium disilicate glass ceramic to study specifically the junction areas. *C. albicans* was incubated in a biofilm reactor at 37 °C with agitation. The biofilm formation was evaluated at 24 and 48 hours. In addition, the morphology of the biofilm was evaluated by scanning electron microscopy.

**Results:**

*C. albicans* developed biofilms on the surface of all materials tested. Cobalt-chromium alloy showed the lowest density of adhered biofilm, followed by zirconia and titanium. Silicone and resin showed up to 20 times higher density of biofilm. A higher biofilm formation was observed when junctions of materials presented micropores or imperfections.

**Conclusions:**

The biofilm formed in the three materials used in the manufacture of abutments and prostheses showed no major differences, being far less dense than in the resins. Two clinical recommendations can be made: to avoid the presence of resins in the subgingival area of implant prostheses and to design prostheses placing cobalt-chromium alloy/ceramic or titanium/ceramic junctions as far as possible from implants.

** Key words:**Dental implants, cobalt-chromium alloy, titanium, zirconia.

## Introduction

Modern implant Dentistry is a predicTable and safe alternative for replacing missing teeth (1). Improvements in treatment protocols and in design and properties of implants and prostheses have drastically reduced initially observed osseointegration-related failures (2). Nowadays, the great challenge for implant dentistry is to reduce the still high rate of late biological complications such as peri-implant mucositis or peri-implantitis (1,2).

Peri-implant mucositis and peri-implantitis are biofilm-related diseases that occur depending on different factors, in which the individual susceptibility plays a major role. The development of these diseases can be triggered or modulated by many additional mechanisms with varying degrees of available evidence (1-3). Morphological and physicochemical characteristics of both implants and prostheses directly influence the formation of biofilm on their surface and subsequently the risk of biological complications (4). Biofilm formation is directly conditioned by the physical and chemical characteristics of the materials (5,6,7). Additionally, saliva contains several substances that can modify surface properties (7). The presence of nutrients and the complexity of microbial interrelations of the hundreds of oral different microorganisms are also factors that can influence in the development of biofilms (8). In addition, there are other factors related to the host that affect the formation and maturation of oral biofilm, such as hygiene degree, systemic conditions or pathologies or tobacco consumption (4).

Properties of the materials employed to manufacture abutments and prostheses can be as important as the properties of the implants, to achieve the most desirable conditions: biocompatibility and resistance to microbial colonization (9). Research on relations between materials used to manufacture dental implants and biofilm development on their surfaces is extensive (1,4). Nevertheless, research employing abutments and prostheses materials is recent and scarce (10).

Abutments and prostheses are in direct contact with soft tissues where peri-implant inflammatory diseases start and afterwards spread to bone (1,3). Microscopic and macroscopic physicochemical and morphological characteristics of the first 2-3 mm over the implant-prosthesis connection are the key areas conditioning treatment success (11,12). This key-area is in direct contact with connective and epithelial tissues and even with the bone, depending on the implant-prostheses connection design. Therefore, the type of material selected for this area and its manufacturing and finishing processes are critical to ensure biocompatibility and infection preclusion.

Titanium (Ti) and cobalt-chromium alloys (Co-Cr), and zirconium dioxide (ZrO2) are among the most commonly used materials in implantology to manufacture definitive abutments or the transmucosal portion of dentures (10). Biofilm formation process on these surfaces have not been fully clarified and some controversies still persist. Hence, new approaches to improve the features of abutments and dentures manufactured with these materials are desirable to ensure biocompatibility, to reduce the subgingival biofilm formation and, subsequently, to minimize the prevalence of biological complications.

In this work we have studied the *in vitro* ability of **Candida* albicans* biofilm formation over materials commonly employed in Dentistry, using a culture model to simulate oral conditions. The main objective was to evaluate the quantitative differences in the colonization and adhesion of *C. albicans* over different materials used to manufacture abutments and dentures. Moreover, we have analyzed the characteristics of the colonization patterns on every different surface by scanning electron microscopy.

## Material and Methods

The fungal strain used for this study was *C. albicans* SC5314 (Berkhout, ATCC® MYA-2876, Manassas, USA). The study was designed conform to the requirements for biosafety level 2 and approved by the Research Ethics Committee for Biological Agents and Genetic Modified Organisms of the University of the Basque Country, UPV/EHU (Reference: M30_2015_248).

- Sample preparation

The materials selected for the study were commercially pure titanium grade II (Ti), yttria-stabilized zirconia (ZrO2), and a cobalt-chromium alloy (Co-Cr). Silicone, nano-hybrid restorative composite and polymethylmethacrylate (PMMA) were also included as materials usually present in oral prostheses. Main characteristics of the materials are summarized in Table 1.

**Figure 1:**
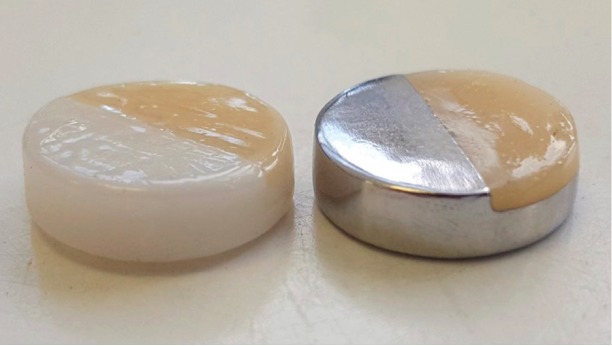
Discs half-covered with Ls2 ceramic prepared to observe the features of the junction area by SEM.

A set of 12 discs of each material was used to quantify the formation of biofilm at 24 h and 48 h and for scanning electron microscopy (SEM) analysis. Additionally, 3 discs, the ones made of Ti, ZrO2 and Co-Cr, were half covered with lithium disilicate (Ls2) glass ceramic (IPS, Ivoclar, Amherst, USA) to study specifically the junction between both materials by SEM (Fig. 1).

**Table 1:**
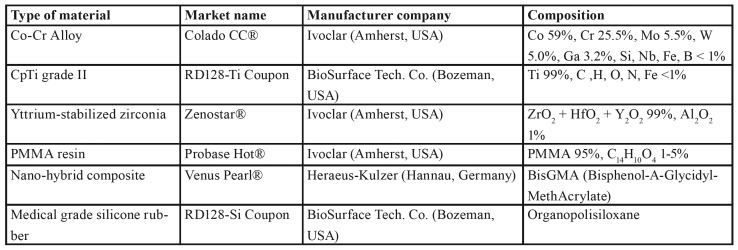
Materials analyzed in the study.

All discs had a diameter of 12.7 mm (0.50 in) and a thickness of 3.8 mm (0.15 in). The Ti disc coupons (RD128-TI) were used as supplied by the provider (Biosurfaces technologies Co., Bozeman, USA). Co-Cr discs were obtained by lost wax casting. Then a finishing and polishing process was applied to them in a dental laboratory, trying to simulate the actual manufacturing process of the implant prostheses. A pre-polishing was performed with carbide finishing burs and increasing grit rubber wheels (Komet, Brasseler, Germany). Final polishing was carried out with buffing wheels and 5/2.5 µm diamond polishing paste (Komet). ZrO2 discs were produced with a 5 axis milling machine (Zenotec®, Ivoclar vivadent, Schaan, Liechtenstein). After milling, ZrO2 discs were sintered at 1,400 °C, glazed and finished with the same buffing wheels and polishing paste used with Co-Cr discs. PMMA and composite discs were manually prepared according to manufacturer's instructions. In these cases, Sof-Lex® Contouring and Polishing Discs (3M, Maplewood, USA) were additionally used in the polish procedure. Silicone was tested in disc coupons (RD128-Si) as provided by the manufacturer (Biosurfaces technologies Co.), without any additional procedure. In the three discs that were partially covered with Ls2 glass ceramic, glazing of the ceramic was performed before polishing both surfaces with the same protocol described for ZrO2 and Co-Cr discs, as the ultimate goal was to reproduce realistic conditions of use of these materials in the mouth. Discs were UV sterilized during 30 min to avoid damage to thermos-sensitive materials such as resins.

- Culture medium preparation

The medium selected for initial *C. albicans* growth was YEPD (yeast extract 1%, peptone 2% and glucose/dextrose 2%). The study of biofilm adhesion and formation was performed in a medium rich in glucose and amino acids: RPMI (Roswell Park Memorial Institute medium) 1640 medium (Sigma-Aldrich, St. Louis, USA) with L-glutamine and without NaHCO2, buffered to pH 7 with 0.165 M morpholinepropanesulfonic acid (Sigma-Aldrich). Saline phosphate buffer (PBS, Sigma-Aldrich) was used for cell cleaning and during the sonication and vortex mixing processes. Sabouraud dextrose agar (Difco Laboratories, Detroit, USA) was used in the counting procedure of the cells detached from the materials.

- Inoculum preparation 

Several colonies of *C. albicans* SC5314 were suspended in 5 ml of YEPD and incubated for 24 h at 37 °C. Cells were then centrifuged for 9 min at 3500 rpm and washed with PBS twice. The suspension was adjusted to 106 cells/ml by counting on a hemocytometer. One ml of the inoculum was mixed to 500 ml of RPMI and added to the bioreactor.

- Bioreactor preparation

CDC Biofilm Reactor® (Biosurfaces technologies Co.) consists on a one-liter glass vessel with a polyethylene lid that supports eight independent and removable polypropylene rods, and with a gas exchange port. Each rod can accommodate three sample discs. The culture medium circulated through the vessel by magnetic stirring. Before starting, the bioreactor and all its components were autoclaved at 121 °C for 15 min. Once the disc samples were assembled on the rods and the lid fixed in aseptic conditions, the culture medium and inoculum were added and incubated at 37 °C with 100-125 rpm agitation for a total time of 24-48 h.

- Collection and analysis of results

To estimate the number of cells adhering to the surface, sample discs were removed from the bioreactor vessel after 24 h and 48 h of incubation and placed into conical tubes with 30 ml of PBS each. Biofilm detachment was carried out by 30 s of vortex mixing, 2 min of sonication (VCX 130, Sonics Materials, USA) at 50% of amplitude followed by 30 s of vortex mixing. Resulting cell suspensions were sequentially diluted and 100 µl of the dilution were inoculated onto Sabouraud agar plates and incubated for 24 h at 37 °C.

Biofilm burden was presented as the mean of the logarithm of colony-forming units (CFU) per cm2 of disc. Density average of biofilm on the discs of each material was calculated, both at 24 h and 48 h. All procedures were conducted in triplicate in two separate days.

- Statistics

Biostatistical analysis was carried out using SPSS 24 statistical software (IBM SPSS Statistics, IBM, Armonk, USA). Kolmogorov-Smirnov and Shapiro-Wilk normality tests were used to determine if the analyzed data had a normal distribution. ANOVA and Tukey's range test were used to analyze differences among means. A value of *p* < 0.05 was considered statistically significant.

- Electron microscopy

In order to analyze the characteristics and patterns of the biofilms formed by *C. albicans* on the surface of the studied materials, three extra sets of discs were prepared following the same methodology. After removing the discs from the coupon holders at 48 h, they were fixed in 2% glutaraldehyde and were sent to the Analytical and High-Resolution Microscopy in Biomedicine Service of the University of The Basque Country UPV/EHU (SGiker) to proceed with the sample preparation for SEM. Briefly, the samples were washed and dehydrated with a series of ethanol solutions. Then they were dried and placed on supports to be covered with gold in an argon atmosphere. The images were obtained with the Hitachi S4800 model microscope, filament voltage 10.0 kV (Hitachi High-Technologies Corporation, Tokyo, Japan).

## Results

Biofilm densities on each material were transformed to a logarithmic basis to simplify data management. According to biofilm development, materials could be divided in two groups. Biofilms developed on Co-Cr, ZrO2 or Ti were significantly less dense than that observed on silicone, nano-hybrid composite or PMMA.

Lowest density of biofilm at 24 h (4.16 ± 0.29 log CFU/cm2 –mean ± standard deviation-) and at 48 h (3.85 ± 0.29 log CFU/cm2) was observed on Co-Cr followed by biofilm on ZrO2 (24 h: 4.58 ± 0.22 log CFU/cm2, 48 h: 4.09 ± 0.11 log CFU/cm²) and on Ti (24 h: 4.76 ± 0.26 log CFU/cm², 48 h: 5.48 ± 0.24 log CFU/cm²). There were no statistical differences between C. albicans biofilm developed on Co-Cr or ZrO2, although less biofilm was formed on Co-Cr than on Ti (*p* = 0.008). ZrO2 showed less biofilm than Ti at 48 h (*p* < 0.001), but similar to Ti at 24 h (*p* = 0.796).

**Figure 2:**
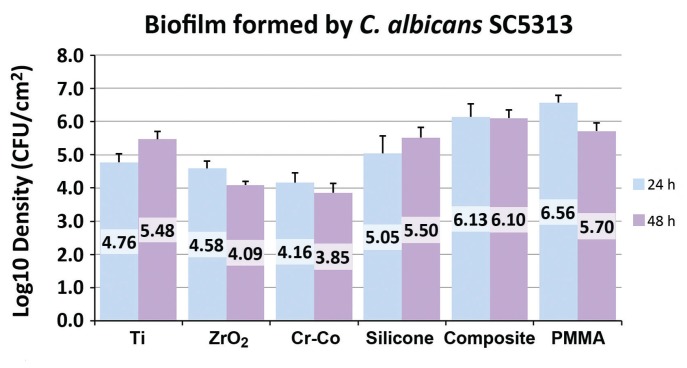
Density of biofilm formed at 24 h and 48 h on each material studied.

The biofilm formed on silicone, nano-hybrid composite or PMMA, had densities up to 20 times higher than on Ti, ZrO2 or Co-Cr. Biofilm on silicone showed lower density (24 h: 5.05 ± 0.52 log CFU/cm², 48h: 5.50 ± 0.32 log CFU/cm²) than nano-hybrid composite (24 h: 6.13 ± 0.39 log CFU/cm², 48 h: 6.10 ± 0.24 log CFU/cm²) or PMMA (24 h: 6.56 ± 0.21 log CFU/cm², 48 h: 5.70 ± 0.27 log CFU/cm²). However, there were no statistical differences between them. All the results are summarized in Fig. 2.

Biofilms developed on Ti showed a significant increase between 24 h and 48 h incubations, while biofilms on Co-Cr and ZrO2 remained similar at both incubation times. Nano-hybrid composite maintained similar levels of biofilm density at both reading times. Conversely, biofilms on silicone increased at 48 h and biofilms on PMMA decreased significantly.

**Figure 3:**
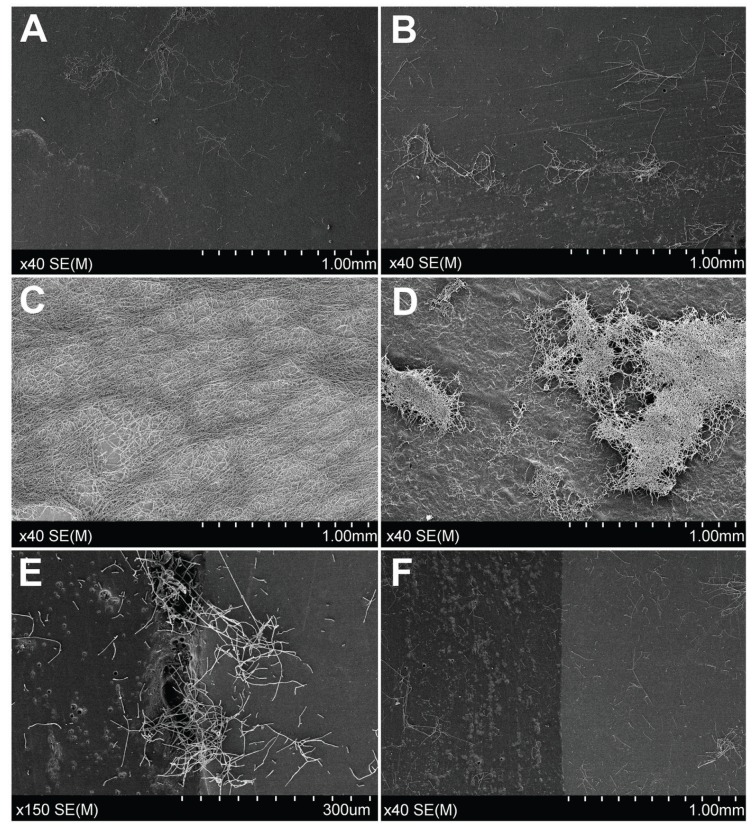
SEM images of biofilm development on different materials and times of incubation: A) ZrO2 at 48 h; B) Co-Cr at 48 h; C) Ti at 48 h; D) PMMA at 48 h. E) SEM images of junction between Co-Cr and Ls2 ceramic: Areas of micropores, imperfections or greater roughness presented more and much denser hyphae clusters attached to the surface; F) SEM images of junction between Co-Cr and Ls2 ceramic: Areas free of defects at the junctions did not show relevant presence of biofilm.

Biofilm formation on Ti, ZrO2 and Co-Cr showed similar patterns and characteristics when observed by SEM at 48 h: On ZrO2 and Co-Cr scattered pseudohyphae clusters of variable sizes and scarce blastoconidia were observed. However, on Ti and PMMA biofilms showed a denser network of hyphae and pseudohyphae where the fungal network almost covered the complete surface of discs (Fig. 3).

When the junction between Ti, ZrO2 or Co-Cr and the Ls2 ceramic was studied, the number of hyphae clusters was remarkable lower on Ls2 ceramic surfaces than on the surfaces of discs of other materials. Very interestingly, more abundant and denser hyphae clusters were attached to the surface in those areas of the junctions where pores, defects or greater roughness were seen. Junction between ZrO2 and Ls2 ceramic was the most homogenous and free of surface irregularities and no more biofilm growth was observed in that specific area.

## Discussion

Oral cavity surfaces are prone to develop biofilms (13). Controlling oral biofilms is an everlasting major concern for clinicians, researchers and dental material manufacturers, since biofilms are involved in short and long term success of implant treatments (3,4). Although modern implantology is predicTable and has high success rates, peri-implantitis prevalence has increased at the same time as the number of implants placed (3,4). All materials used in dental implantology should ideally meet two premises: high biocompatibility and high resistance to microbial colonization. To improve these conditions and to develop new strategies to prevent biological and mechanical complications, it is essential to focus research on understanding the processes of adhesion and colonization on the different materials employed in implantology. *In vivo* studies of oral diseases are frequently associated with ethical concerns; that is why diverse artificial biofilm models are usually used to reproduce oral conditions (14). Some models have tested biofilm formation on implants surfaces, comparing different materials or/and surface treatments (5,15). However, biofilm on transmucosal abutments or on the transmucosal portion of implant prostheses has been scarcely studied (16,17), although in most cases peri-implant pathologies begin in the soft and hard tissues in direct contact with these materials to further progress to bone (1,16). In the current study, knowledge about biofilm formation over the materials employed to manufacture abutments and dentures and not only over implants have been widened.

Mono-species artificial biofilm model systems are of great importance, when performed under controlled conditions, to understand the surface-microorganism interactions, to evaluate the effects of surface modifications and to detect antimicrobial activity or microbial growth induction of the materials. We used a widely employed biofilm model to test different materials related to medical devices (18,19). To the best of our knowledge, the bioreactor-based methodology has not been used before to test the biofilm formation over abutment/prosthetic materials. In contrast to other previous works (5,6,20), we prepared the sample discs following the protocols developed in the dental laboratories in order to be able to come to conclusions to the clinical practice.

In our study, the least dense biofilms were developed on Co-Cr and ZrO2. In spite of the differences in the methodology employed our observations on the favorable properties of the ZrO2 are in order to those of Li *et al*. (21). These authors analyzed *C. albicans* biofilm formation on seven materials commonly used in implantology by SEM. These seven materials that were studied uncoated and saliva-coated included type-I collagen coated polystyrene, hydroxyapatite, ZrO2, cp grade II Ti, acrylic resin, polyethylene terephthalate (PET), Co-Cr alloy and gold-silver-palladium alloy. From all of them, ZrO2 showed the lowest susceptibility to adhesion either uncoated or saliva-coated. Furthermore, other studies have been designed to evaluate the adherence of *C. albicans* to diverse implant surfaces and not specifically the materials used to manufacture prostheses (5,22,23). In this way, Bürgers *et al*. (5) evaluated yeast adhesion to three differentially textured commercialized Ti implants and one ZrO2 implant. They found the lowest formation of biofilm on sand-blasted Ti surface implants. ZrO2 implants showed similar levels of *C. albicans* biofilm formation to machined Ti and sand-blasted and acid-etched Ti implants. They also observed that salivary mucin could serve as a receptor for yeast adhesion whereas albumin could act blocking the adhesion process. This effect of salivary mucin on the development of biofilms over dental materials has also been observed *in vitro* by Li *et al*. (21).

Co-Cr alloys have a wide range of applications in dentistry owing to their good mechanical properties, good biocompatibility and lower price (24). However, there are disadvantages, such as their higher corrosion in acidic environments, the difficulties to get an optimal finishing and polishing, the limited knowledge of their longevity and a lower biocompatibility than precious alloys (24,25). Co-Cr abutments and prostheses can be obtained by casting or milling, following different manufacture procedures and the frequency of its use varies greatly among different countries (24-26). This could be one of the factors that explains the controversial results when studying biofilm formation on Co-Cr. Other differences could be probably due to differences in the preparation of the samples, microbiological procedures and the measurement of biofilm formation. Surprisingly, Co-Cr discs in our study showed the least amount of biofilm formation, contrary to what was observed by Souza *et al*. (27). They evaluated and compared the density and the morphological aspects of biofilms on Co-Cr alloy, feldspar-based porcelain, cpTi grade IV and yttrium-stabilized zirconia. They detected a higher accumulation of oral biofilms on Co-Cr based materials than that on Ti, ZrO2, or porcelain, especially after 48 h. Nevertheless, the methodology used in their study was different to the one followed in this study, based on multi-species static culture model using different sample finishing and polishing protocols. Jordan *et al*. (28) investigated the adherence to electropolished Co-Cr produced by selective laser melting and to milled Ti grade V (Ti-6Al-4V) of periodontal relevant bacterial species. Interestingly, they observed by confocal laser scanning fluorescence microscopy a higher adherence of **Porphyromonas gingivalis** and *Fusobacterium nucleatum* on Co-Cr than on Ti.

Ti and its alloys are undoubtedly the most studied materials in implant dentistry regarding biofilm formation. Our study and other studies showed that its resistance to adhesion is generally similar to that of ZrO2 and Co-Cr, although some noTable differences have been noticed (7,15,16). Heterogeneity of results is probably due to diverse factors as the nature of the samples, including the type of Ti employed (according to ASTM international standards II, IV or V), surface treatments and coatings, finishing and polishing protocols or sterilization protocols. In turn, other differences seem to be directly related to the design of the studies. A different approach to evaluate this issue was developed by Bevilacqua *et al*. (15) that, using confocal laser scanning microscopy, compared *in vivo* and *in vitro* the biofilm formation on Ti surfaces of different roughness. These authors observed that quantitative differences among different roughness surfaces were not predictive of microbial colonization rates *in vivo*.

ZrO2 and Ti surface modifications have direct effect on biofilm formation. Thus, *C. albicans* biofilm formation could be reduced by techniques such as silica-coating or silanization (22). Hydrophobic and hydrophilic strains of *C. albicans* are able to adhere to a plethora of ligands through complex mechanisms colonizing a great variety of oral niches (29). Hence, wettability is a very important factor when designing biofilm formation studies and also when interpreting the results of previous research.

Resins have a low resistance to biofilm formation compared to other materials (21). In our study nano-hybrid composite or PMMA showed worse behaviour than metals and ceramics which supports the observations of Li *et al*. (21). An obvious clinical application of this observation could be to avoid or limit their presence in the subgingival area when designing hybrid and temporary prostheses. Multispecies biofilm formation models on new materials such as polyetheretherketone (PEEK), compared to PMMA, Ti or ZrO2, have shown promising results as observed by Hahnel *et al*. (20). However clinical studies are necessary to confirm these preliminary results.

Another important factor to consider when comparing results in relation to the formation of biofilms is the sterilization method used with the samples. Han *et al*. (23) investigated the effect of four different sterilization methods on **P. gingivalis** and *Staphylococcus aureus* biofilm formation. The surface free energy, surface chemistry and wettability were differently affected by sterilization methods. The dry heat sterilization treatment reduced the formation of biofilms, while irradiations with ultraviolet light (UV) or X rays increased their development. Our UV-based sterilization protocol was exactly the same for all the samples to avoid bias. The choice of this method was based on the fact that some materials such as resins could be damaged by other methods.

Differences observed using electron microscopy in the junction zones between Ti / Co-Cr / ZrO2 and Ls2 ceramic should be highlighted. The junction between Co-Cr and Ls2 ceramic showed the highest rate of irregularities, crevices, ridges and micropores. Micropores were areas where highest biofilm density was observed. Obviously, the study discs had a flat surface which facilitates finishing and polishing, and had been manufactured with extreme care. Real junctions in crowns and bridges are frequently not so easy to finish, and contain even more micropores and imperfections. In an attempt to prevent peri-implantary diseases, and in light of these results, it would be desirable to design abutments and prostheses maintaining metal-ceramic junctions as far as possible from implants. On the contrary, the junction ZrO2-Ls2 ceramic was the most homogenous and nearly free of surface irregularities. No further growth of biofilm was observed in this type of junction. This property could be of interest when designing prostheses, especially in the aesthetic area.

## Conclusions

The lowest dense biofilms were developed on Co-Cr and ZrO2 discs. However, there were no major differences between them and Ti. Biofilm formed was up to 20 times greater on resins. Junction areas in the sample discs accumulated densest biofilms except in the ZrO2-Ls2 junction. Two clinical recommendations arise from current results: The presence of resins in the subgingival area of implant prostheses should be avoided, and in the prostheses design Cr-Co / Ti and ceramic junctions should be placed as far as possible from implants.
